# Poor survival of Methicillin‐resistant *Staphylococcus aureus* on inanimate objects in the public spaces

**DOI:** 10.1002/mbo3.308

**Published:** 2015-10-26

**Authors:** Hisanori Domon, Yoshio Uehara, Masataka Oda, Hiromi Seo, Noriko Kubota, Yutaka Terao

**Affiliations:** ^1^ Division of Microbiology and Infectious DiseasesNiigata University Graduate School of Medical and Dental SciencesChuo‐kuNiigataJapan; ^2^ Department of Life Science Reserch CenterNagano Children's HospitalToyoshinaAzuminoNaganoJapan; ^3^ Kochi Medical SchoolKochi UniversityOkocho KohasuNankokuKochiJapan

**Keywords:** Desiccation tolerance, inanimate objects, multilocus sequence typing, public spaces, *Staphylococcus aureus*.

## Abstract

We investigated the prevalence of *Staphylococcus aureus* on shopping baskets in Osaka Prefecture, Japan. Multilocus sequence typing was performed to determine the genotypes of *S. aureus* isolates, and then a polymerase chain reaction method was used to detect staphylococcal enterotoxins and antibiotic resistance genes. In addition, desiccation tolerance of *S. aureus* isolates was evaluated in vitro. Forty‐six (6.2%) *S. aureus* isolates were collected from 740 shopping baskets, though only one MRSA strain was identified. In multilocus sequence typing findings, ten sequence types and 24 singletons were classified, which were divided into ten clonal complexes and six singletons. The most frequent staphylococcal enterotoxin gene was *seg* (30.4%). Our *in vitro* findings demonstrated that 70% of the *S. aureus* isolates, including the MRSA strain, became undetectable at 12 h after desiccation at an appropriate cell density, while the others remained viable for up to 24 h. Thus, it is difficult for MRSA organisms to survive on dry surfaces found in public areas. We speculated that inanimate objects in the community are unlikely to be a potential source for transmission of MRSA and that *S. aureus* on such objects outside of hospital settings is not a public health threat.

## Introduction

Methicillin‐resistant *Staphylococcus aureus* (MRSA) is a bacterium known to be resistant to not only *β*‐lactam antibiotics, but also other antibiotics, and a major cause of healthcare‐associated infections, such as pneumonia, bacteremia, skin infection, and postoperative wound infection. Such MRSA infections are accompanied by significant morbidity and mortality, particularly in immunosuppressed patients (Baird and Hawley 2000). For this reason, many countries have taken countermeasures against MRSA. As a result of an effective infection control policy termed “Search and Destroy”, the percentage of *S. aureus* bacteremia cases caused by MRSA is lower than 1% in the Netherlands and Scandinavian countries (Tiemersma et al. 2005), whereas higher rates of MRSA have been reported in Japan and the United States. In fact, MRSA strains have been found to account for 70% and 30–60% of nosocomial *S. aureus* bloodstream infections, respectively, in those countries (Boyce et al. 2005). It is also generally considered that excessive use of antibiotics can spread and encourage further antibiotic‐resistant strains, making physicians hesitant to prescribe those drugs.

*Staphylococcus aureus* is usually transmitted by direct person‐to‐person contact (Davis et al. 2012), though other indirect transmission routes may include environmental exposure to aerosol, settled dust, and inanimate objects. Therefore, over the past several decades, a number of studies have focused on the desiccation tolerance of *S. aureus* (Chaibenjawong and Foster 2011). In this regard, it has been shown that *S. aureus* remains viable on dry surfaces for periods of at least 1 week and up to 3 years (Neely and Maley 2000; Wagenvoort et al. 2000; Chaibenjawong and Foster 2011). Although several reports have demonstrated survival of *S. aureus* in household environment and healthcare settings, scant attention has been given to spreading of these organisms, including MRSA, throughout the community (Davis et al. 2012).

Shopping carts and baskets used in supermarkets are exposed to potential bacterial contamination through contact with customers’ hands and a variety of foods (Mizumachi et al. 2011). In addition to the high prevalence of MRSA in healthcare settings, those carts and baskets might also be frequently contaminated with MRSA. However, to date, MRSA contamination on environmental surfaces is not considered to be a public health concern in Japan because of limited evidence.

In the present study, we investigated the prevalence of *S. aureus*, including MRSA, on shopping baskets obtained from several supermarkets in Japan. Our findings demonstrate that MRSA is scarcely detectable on the surface of those baskets. Furthermore, in contrast to previous reports, we also found that MRSA organisms at a probable density on dry surfaces of inanimate objects do not survive well under laboratory conditions. Our results provide better understanding of how MRSA may spread in the community, which is important for treatment and prevention of transmission.

## Experimental Procedures

### Bacterial isolates and culture conditions

Bacterial collection was performed at four supermarkets in Osaka Prefecture, Japan, according to previously described methods (Mizumachi et al. 2011). The handles of shopping baskets were swabbed with cotton swabs and dipped in 0.85% saline. Bacteria on the swabs were then inoculated onto Staphylococcus Medium 110 agar plates (Becton Dickinson & Co, Sparks, MD) supplemented with 5% egg yolk (Kyokuto, Tokyo, Japan) in an aseptic manner. The plates were transported back to the laboratory in a chilled condition and cultured for 48 h at 37°C. *S. aureus* was identified by using api‐Staph reagents (Sysmex‐Biomerieux, Tokyo, Japan), and polymerase chain reaction (PCR) assays for the *femA* and *femB* genes (Omoe et al. 2005).

### DNA extraction and PCR amplification

DNA extraction from the organisms was performed according to a previously described method (Mizumachi et al. 2011). Multiplex PCR was performed to determine staphylococcal enterotoxins (SEs) (*sea*,* seb*,* sec*,* sed*,* see*,* seg*,* seh*,* sei*,* selj*,* sek*,* sell*,* sem*,* sen*,* seo*,* sep*,* seq*,* selr*), *tsst‐1*, and antibiotic resistance genes [*blaZ*,* ermA*,* ermC*,* aac* (*6’*)*‐aph* (*2”*), *mecA*]. The *S. aureus* strains N315, Mu50, MW2, and TY129 were used as controls (Mizumachi et al. 2011).

### Multilocus sequence typing

Multilocus sequence typing (MLST) was performed as previously described (Enright et al. 2000). Briefly, seven housekeeping genes (*arcC*,* aroE*,* glpF*,* gmk*,* pta*,* tpi*,* yqiL*) in *S. aureus* were amplified by PCR and the DNA sequences were determined. Alleles at the seven loci were assigned by comparing the sequences at each to those of known alleles in the *S. aureus* MLST database (http://saureus.mlst.net/), then each allelic profile was defined based on sequence type. The eBURST algorithm was used to assign MLST clonal complexes (CCs) (http://eburst.mlst.net/) and a dendrogram was constructed from the pairwise differences in allelic profiles, using MEGA 3.1 software (Kumar et al. 2004).

### Desiccation tolerance assay of MRSA and MSSA

Desiccation tolerance assays were performed with five MRSA strains (NILS1‐5) and four methicillin‐susceptible *S. aureus* (MSSA) strains (NILS6‐9), both of which were isolated from severe *S. aureus* pneumonia cases and kindly provided by Dr. Yagi (Nagoya University Graduate School of Medicine). Other MSSA strains (B2, B15, B18, B49) were isolated from shopping baskets tested in the present study. All *S. aureus* isolates were grown overnight (20 h) in brain–heart infusion (BHI) broth, then cells were washed once by centrifugation at 6000 × g for 20 min, resuspended in distilled water, and adjusted for the initial inoculum [approx. 2–5 × 10^6^ colony forming units (CFU) or 2–5 × 10^4^ CFU per 10 *μ*L]. CFU of *S. aureus* was determined by plating on Staphylococcus medium 110 agar plates for baseline measurement. Ten microliters of diluted suspension was placed into 1.5‐mL polypropylene tubes followed by air drying. Cells were resuspended in 1 mL of PBS followed by sonication (5 min) and plated on Staphylococcus Medium 110 agar plates at each time point to determine rates of viability.

## Results

### Low prevalence of MRSA on the handle of shopping baskets

A total of 740 handles of handheld shopping baskets were sampled with *S. aureus* strains isolated from 46 (6.2%) of those samples (Table 1). Among all of the *S. aureus* isolates, one strain possessed the *mecA* gene (2.1%). Table S1 shows the prevalence of *S. aureus* in the present study as well as two other previous studies, which examined 289 nasal swab samples obtained from healthy subjects in the same region (Mizumachi et al. 2011) and approximately 1.5 million clinical samples from Japanese patients (Japan nosocomial infection surveillance, 2012. http://www.nih-janis.jp/report/index.html).

**Table 1:** Prevalence of *S. aureus* isolated from shopping basket handles

	Shopping baskets (*n* = 740)	Prevalence (% [95% CI])
Total SA isolates	46	6.2% (4.7–8.2)
MSSA isolates	45	6.1% (4.6–8.0)
MRSA isolates	1	0.1% (0.0–0.8)
MRSA/SA (%)		2.1% (0.4–11.3)

SA, *Staphylococcus aureus*; MRSA, methicillin‐resistant *Staphylococcus aureus*; MSSA, methicillin‐sensitive *Staphylococcus aureus*.

### Distribution of toxin genes and antibiotic resistance genes harbored by *S. aureus* isolates

**Figure 1:**
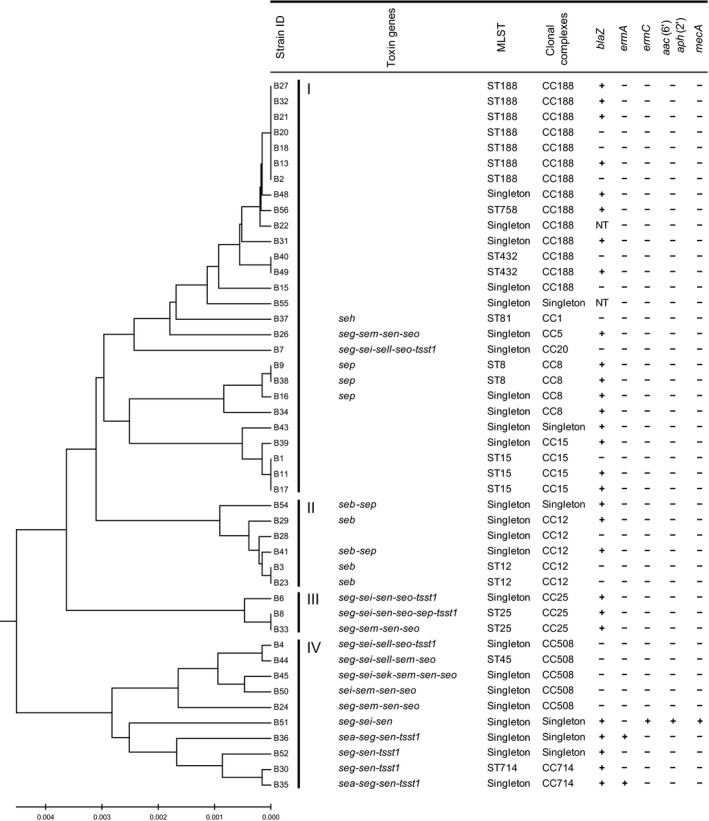
Genomic profiling of *Staphylococcus aureus* isolates on shopping baskets. Forty‐six *S. aureus* isolates were resolved by multilocus sequence typing into 10 clonal complexes. A dendrogram was constructed from the pairwise differences in their allelic profiles. Results of PCR assays for toxin and antibiotic resistance genes are summarized to the right of the cluster. NT: not tested.

*S. aureus* produces a wide variety of toxins including SEs, which demonstrated emetic activity, SE‐like (SEl) toxins, which were not emetic in a primate model or not tested, and toxic shock syndrome toxin (TSST‐1) (Hu and Nakane 2014). SEs and SEls have been subdivided into classical (SEA to SEE) and new (SEG to SElX) types. Therefore, we next examined the distribution of toxin genes in *S. aureus* isolates using a multiplex PCR method (Table 2). The most frequent toxin gene was *seg* (30.4%), followed by *sen* (26.1%), *seo* (21.7%), *sei* (17.4%), and *tsst‐1* (17.4%). On the other hand, none of the isolates harbored *sec*,* sed*,* see*,* selj*,* seq*, or *selr*. Isolates harboring the *seg* gene were also positive for other toxic genes, especially *sen* (78.6%), *seo* (64.3%), *tsst‐1* (57.1%), and *sei* (50.0%) (Fig. 1). In addition, the prevalence of toxin genes varied among the ten CCs (Table 3, Fig. 1). In this study, CC188 was most common (30.4%), following by CC12 (10.9%) and CC508 (10.9%). CC188 and CC15 (8.7%) isolates harbored no toxin genes investigated in this study. In total, 22 isolates (47.8%) harbored no toxin genes. Although CC12 isolates were highly positive for *seb*, a classical type of *se* gene, others were not, with the exception of one isolate, which was classified as a singleton. CC508 isolates harbored multiple types of new *se* genes, such as *seo* (100%), *seg* (80%), *sei* (80%), and *sem* (80%). As for antibiotic resistance genes, 64% of the *S. aureus* isolates possessed the *β*‐lactamase resistance gene *blaZ*. None harbored other antibiotic resistance genes, with the exception of B51, B36, and B35, which were *mecA*‐ or *ermA*‐positive isolates.

**Table 2:** Distribution of staphylococcal toxin genes among the isolates from shopping baskets

	Total	*sea*	*seb*	*sec*	*sed*	*see*	*seg*	*seh*	*sei*	*selj*	*sek*	*sell*	*sem*	*sen*	*seo*	*sep*	*seq*	*ser*	*tsst‐1*
Shopping baskets (*n* = 740)	46	2	5	–	–	–	14	1	8	–	1	3	6	12	10	6	–	–	8
Percentage		4.3	10.9	–	–	–	30.4	2.2	17.4	–	2.2	6.5	13.0	26.1	21.7	13.0	–	–	17.4

**Table 3:** Distribution of Staphylococcal toxin genes in each clonal complex

Toxin genes	CC1*n* = 1	CC5*n* = 1	CC8*n* = 4	CC12*n* = 5	CC15*n* = 4	CC20*n* = 1	CC25*n* = 3	CC188*n* = 14	CC508*n* = 5	CC714*n* = 2	Singleton*n* = 6	Total
*sea*	–	–	–	–	–	–	–	–	–	1	1	2
*seb*	–	–	–	4	–	–	–	–	–	–	1	5
*sec*	–	–	–	–	–	–	–	–	–	–	–	–
*sed*	–	–	–	–	–	–	–	–	–	–	–	–
*see*	–	–	–	–	–	–	–	–	–	–	–	–
*seg*	–	1	–	–	–	1	3	–	4	2	3	14
*seh*	1	–	–	–	–	–	–	–	–	–	–	1
*sei*	–	–	–	–	–	1	2	–	4	–	1	8
*selj*	–	–	–	–	–	–	–	–	–	–	–	–
*sek*	–	–	–	–	–	–	–	–	1	–	–	1
*sell*	–	–	–	–	–	1	–	–	2	–	–	3
*sem*	–	1	–	–	–	–	1	–	4	–	–	6
*sen*	–	1	–	–	–	–	3	–	3	2	3	12
*seo*	–	1	–	–	–	1	3	–	5	–	–	10
*selp*	–	–	3	1	–	–	1	–	–	–	1	6
*seq*	–	–	–	–	–	–	–	–	–	–	–	–
*ser*	–	–	–	–	–	–	–	–	–	–	–	–
*tsst‐1*	–	–	–	–	–	1	2	–	1	2	2	8

CC: clonal complex.

We produced a dendrogram (Fig. 1) showing the genetic diversity of the present *S. aureus* isolates and grouped them into four clusters. Cluster I mainly consisted of CC188, CC8, and CC15, which were negative for toxin genes except for CC8 harboring *sep*, Cluster II consisted of CC12 harboring *seb*, and Cluster III, and IV consisted of CC25, CC508, and CC714 harboring multiple toxin genes, including *seg*. The MRSA and *ermA*‐positive isolates belonged to cluster IV.

### Desiccation tolerance of *S. aureus* isolates on inanimate object

**Figure 2:**
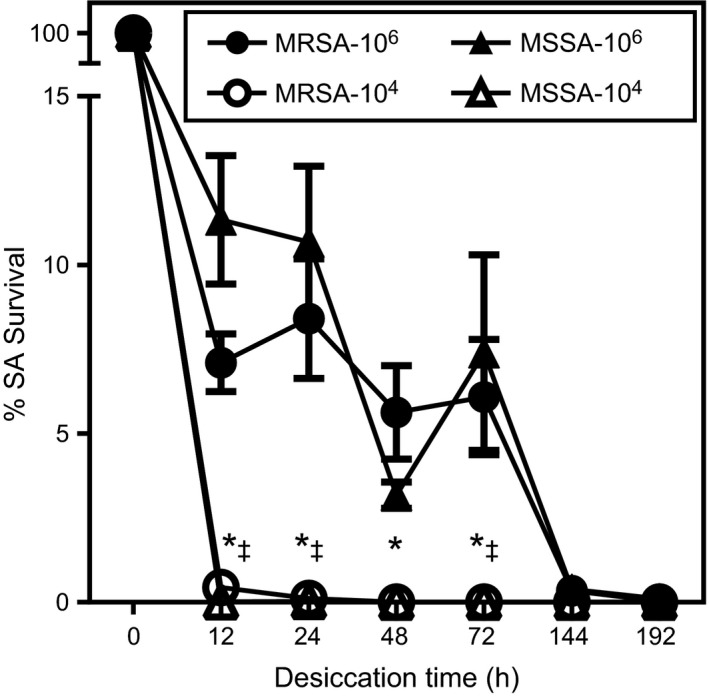
Survival of Methicillin‐resistant *Staphylococcus aureus* (MRSA) on dry surface and maintenance of antibiotics resistance in long‐term culture. Five MRSA and eight MSSA, methicillin‐sensitive *Staphylococcus aureus* (MSSA) isolates with two different cell densities were placed into polypropylene tubes followed by air drying. Viable bacteria were counted after culturing on agar plates at each time point. Each data point represents the mean ± SE. Data were evaluated using two‐way ANOVA and Tukey's multiple comparisons test. *Statistically significant as compared with higher cell density group within the MRSA group, *P* < 0.01. ^‡^Statistically significant as compared with higher cell density group within the MSSA group, *P* < 0.01.

We also examined the desiccation tolerance of five MRSA clinical isolates and eight MSSA isolates (Fig. 2A). At higher cell density (2–5 × 10^6^ CFU per 10 *μ*L), all MRSA and MSSA isolates survived for at least 6 days, with the average rate on day six shown to be 0.34 ± 0.16% and 0.38 ± 0.20%, respectively. On day eight, one isolate in each group was undetectable. Surprisingly, all MRSA and MSSA isolates at a lower cell density (2–5 × 10^4 ^CFU per 10 *μ*L) were undetectable by day two. Furthermore, three of five MRSA isolates and six of eight MSSA isolates were undetectable as soon as 12 h after desiccation.

## Discussion

Few studies have isolated MRSA organisms from samples obtained in community settings and showed their molecular characteristics. In the present study, the isolation frequency of MRSA from inanimate objects was very low, at least when obtained in a dry environment. Same results were observed in additional bacterial collection at 20 supermarkets in Niigata Prefecture, Japan. Sixty‐two (3.0%) *S. aureus* strains, including one (1.6% among all of *S. aureus* isolates) *mecA*‐positive MRSA, were found in a total of 2060 handles of shopping baskets (Uehara, unpubl. data). The prevalence of *S. aureus* on shopping baskets was lower as compared with two other previous studies, which examined nasal swab samples obtained from healthy subjects (Mizumachi et al. 2011) and clinical samples from Japanese patients (Japan nosocomial infection surveillance, 2012. http://www.nih-janis.jp/report/index.html). Also, the percentage of MRSA isolates among all *S. aureus* isolates in our study was lowest. In that latter study, more than 50% of *S. aureus* organisms clinically isolated from Japanese patients were MRSA, whereas MRSA were rarely isolated from the shopping basket samples. These findings suggest that inanimate objects are unlikely to be a potential source for transmission of MRSA outside of hospital settings.

Although it remains unclear whether development of antibiotic resistance by staphylococci organisms occurs from acquisition of resistance‐related genes, healthcare settings are known to be of high risk because of routine antibiotic selection pressure and a higher prevalence of MRSA. In Japan, unlike the Netherlands and Scandinavian countries, there are no national guidelines for prevention of MRSA in relation to hospital admission screening of patients or screening of healthcare workers (Kramer et al. 2010). It is readily apparent that MRSA can spread from a healthcare setting into the community by patients after discharge from the hospital or healthcare workers, as MRSA colonization has been reported to frequently occur among households members of a patient with nosocomial‐acquired MRSA (Calfee et al. 2003). If indirect transmission routes such as inanimate objects in addition to direct person‐to‐person contact were involved in the spread of MRSA throughout the community, then the prevalence of MRSA among community members should be higher than the current status.

*S. aureus* is a hardy microorganism that resists various types of dry environments, including the surface of inanimate objects thought to be fomites. Previous studies have reported the survival of *S. aureus* on polyethylene for 90 days (Neely and Maley 2000), sterile packaging for 266 days (Dietze et al. 2001), screw top bottles for 318 days (Wagenvoort et al. 2000), and polypropylene for more than 1097 days (Hu and Nakane 2014). However, it seems that those researchers achieved staphylococcal survival by optimizing the experimental conditions in a competition for longevity. Notably, those reports used staphylococcal strains that had a high level of resistance against desiccation or high bacterial density of 10^7^–10^9^ CFU. Although those findings were clear and consistent when obtained under laboratory conditions, they might not accurately reflect the survival of *S. aureus* in the community. If *S. aureus* can survive for more than a week on inanimate objects, then the organisms would be expected to accumulate on shopping baskets and the prevalence rate should be >30%, in association with nasal carriage of *S. aureus* in healthy subjects in Japan (Mizumachi et al. 2011). In addition, it has been reported that the density of total aerobic bacteria on hands is approximately 2–3 × 10^6 ^CFU (McGinley et al. 1988), thus 10^7^–10^9^ CFU of *S. aureus* is unlikely to be an adequate amount for transmission from hands to inanimate objects. The low bacterial density of 10^4^–10^5^ CFU shown in the present study seems to be reasonable for elucidating the desiccation tolerance of *S. aureus* in the community.

In contrast to decreased *S. aureus* survival at a low bacterial density under laboratory conditions and the low prevalence of MRSA (0.1%) on shopping baskets, 6.1% of the shopping baskets tested were colonized with MSSA, which may be related to the prevalence of MSSA in healthy individuals (29.4%) and frequent use of the baskets in a single day (Mizumachi et al. 2011). It is possible that an individual shopping basket is used by an MSSA carrier at least once a day and transmitted MSSA can survive for at least 24 h. Median *S. aureus* CFU per handle of contaminated basket was 2 (interquartile range 1–5), which may suggest that *S. aureus* is going extinct. On the other hand, shopping baskets are not frequently exposed to MRSA as compared with MSSA because the prevalence of MRSA in healthy individuals is 2.1% (Mizumachi et al. 2011). In addition, it has been reported that MRSA carriers are usually colonized with one or more MSSA strains (Mongkolrattanothai et al. 2011), thus the bacterial density of MRSA transmitted to shopping baskets along with other MSSA strains at the same time may be relatively lower than that of a single strain. In addition, our desiccation tolerance assay indicates that both MRSA and MSSA strains are sensitive to dry environment with the similar level. Taken together, the lower prevalence and the lower colonization density of MRSA in healthy individuals may lead MRSA to lack persistence on inanimate objects in a community setting.

The possibility of inanimate objects as fomites in healthcare settings has been documented. In this context, many studies have suggested that hospital equipment and mobile communication devices are contaminated with MRSA (0–14%) (Brady et al. 2009; Kei and Richards 2011). Inanimate objects may act as a route of MRSA transmission, at least in healthcare settings, as both inpatients and healthcare workers may be colonized with MRSA (Gomes et al. 2014), hospital equipment can be repeatedly exposed to MRSA each day, and MRSA can be sustained on a dry surface when contained in pus or blood (Tolba et al. 2007). In contrast, those conditions are unlikely to be observed in the community.

MLST analysis revealed ten sequence types (STs) and 24 singletons, and the STs were classified into the following ten CCs and six singletons: CC188 (30.4%), CC12 (10.9%), CC508 (10.9%), CC8 (8.7%), CC15 (8.7%), CC25 (6.5%), CC714 (4.3%), CC1 (2.2%), CC5 (2.2%), and CC20 (2.2%). A recent study demonstrated that *S. aureus* isolates obtained from a Japanese healthy population on nasal swabs mainly consisted of CC508 (18.8%), CC15 (12.8%), CC188 (12.5%), CC5 (8.5%), CC8 (8.2%), and CC12 (8.2%), whereas isolates from staphylococcal food poisoning (SFP) specimens mainly consisted of CC81 (54.8%) (Sato'o et al. 2014). The same study reported that CC81 harboring the *sea* gene was the major lineage of the *S. aureus* SFP isolates, which produced a higher level of SEA as compared with other CC types harboring *sea*. Also, the most frequent enterotoxin genes found in isolates from those nasal swab samples were *seg*,* sei*,* sem*,* sen*, and *seo*, which were also frequently found in *S. aureus* isolates in the present shopping basket specimens. Together with the present findings, it has been shown that dominant CC types and enterotoxins of *S. aureus* isolates closely resemble those of *S. aureus* nasal swab isolates from healthy Japanese individuals. On the other hand, CC81 was not identified in our study. Furthermore, MLST analysis found similarities between the *S. aureus* isolates obtained from shopping baskets and those from nasal swabs of employees of the same supermarket (data not shown). Therefore, it is unlikely that most *S. aureus* isolates on shopping baskets cause SFP.

In conclusion, our findings demonstrate that highly pathogenic *S. aureus* organisms, including MRSA and MSSA, which cause healthcare‐associated infections and SFP, respectively, are rarely isolated from inanimate objects in the community, because of poor survival on a dry surface for more than 24 h. They also suggest that MRSA is an anomalous bacterium that cannot survive in the absence of antibiotic selective pressure or outside of hospital settings. Furthermore, *S. aureus* isolates on shopping baskets are likely to be transmitted by healthy nasal carriers. On the other hand, it is also known that nasal carriers of *S. aureus* have an increased risk of acquiring a related infection (Wertheim et al. 2005). Also, it has been reported that shopping carts may be contaminated with *Salmonella* and *Campylobacter* (Patrick et al. 2010). Therefore, it is recommended that all supermarkets routinely clean shopping baskets and carts, or provide hand sanitizers and sanitary wipes to their customers. Additional studies are needed to further explore the possibility of transmission and colonization of *S. aureus* from inanimate objects to healthy individuals.

## Conflicts of Interest

We declare that we have no conflicts of interest.

## Supporting information


**Table S1.** Prevalence of *S. aureus* isolated from shopping baskets, healthy subjects, and patients.
