# Supplementary material for: Poor survival of Methicillin‐resistant Staphylococcus aureus on inanimate objects in the public spaces
Source: Microbiologyopen. 2015 Oct 26;5(1):39–46. doi: 10.1002/mbo3.308 (PMC4767431; doi:10.1002/mbo3.308)
Supplement: Supplementary file 1 — Table S1. Prevalence of S. aureus isolated from shopping baskets, healthy subjects, and patients. [file MBO3-5-039-s001.docx]

Supplementary Table S1. **Prevalence of *S. aureus* isolated from shopping baskets, healthy subjects, and patients.**

|  | Shopping baskets (%) (n = 740) | Healthy subjects in Japan (%)  ([Mizumachi et al., 2011](#_ENREF_15)) (n = 289) | Patients in Japan (%)  ( http://www.nih-janis.jp/report/index.html) (n = 1453969) |
| --- | --- | --- | --- |
| Total SA isolates | 46 (6.2%) | 91 (31.5%)  85 (29.4%) | 221239 (15.2%) |
| MSSA isolates | 45 (6.1%) |  | 104030 (7.1%) |
| MRSA isolates | 1 (0.1%) | 6 (2.1%) | 117209 (8.1%) |
| MRSA / SA (%) | 2.1% | 6.6% | 53.0% |

SA: *Staphylococcus aureus*

MRSA: meticillin-resistant *Staphylococcus aureus*

MSSA: meticillin-sensitive *Staphylococcus aureus*
